# Validation of a Simple HPLC–UV Method for the Determination of Monomers Released from Dental Resin Composites in Artificial Saliva

**DOI:** 10.3390/mps3020035

**Published:** 2020-05-03

**Authors:** Elisavet-Ioanna Diamantopoulou, Orfeas-Evanggelos Plastiras, Petros Mourouzis, Victoria Samanidou

**Affiliations:** 1 Department of Chemistry, Laboratory of Analytical Chemistry, Aristotle University of Thessaloniki, 54124 Thessaloniki, Greece; diamante@chem.auth.gr (E.-I.D.); orfeplas@hotmail.com (O.-E.P.); 2 Department of Dental Tissues Pathology and Therapeutics, Division of Operative Dentistry, Faculty of Dentistry, Aristotle University of Thessaloniki, GR-54124 Thessaloniki, Greece; p.mourouzis@gmail.com

**Keywords:** validation, artificial saliva, bisphenol-A (BPA), bisphenol A glycerolate dimethacrylate (BIS-GMA), dental composites, CAD/CAM materials, dimethacrylate monomers, HPLC, triethylene glycol dimethacrylate (TEGDMA), urethane dimethacrylate (UDMA)

## Abstract

Bisphenol-A (BPA), bisphenol A glycerolate dimethacrylate (Bis-GMA), triethylene glycol dimethacrylate (TEGDMA), and urethane dimethacrylate (UDMA) are organic monomers that can be released from dental composites into the oral cavity. Over specific concentrations, they can act as endocrine disruptors or cause toxic effects. The purpose of this work is to develop and validate an analytical method to determine BPA, Bis-GMA, TEGDMA, and UDMA monomers released from synthetic dental resins in artificial saliva. The method was validated before its application to new hybrid ceramic materials used in computer-aided design and computer-aided manufacturing (CAD/CAM) restorations to determine the release of monomers in various time intervals (e.g., 24 h, and 7, 14, 30, and 60 days), both in methanolic solutions, as well as in artificial saliva. Chromatographic analysis was performed isocratically on a Perfect Sil Target ODS-3 analytical column (250 mm × 4.6 mm, 5 µm) with CH_3_CN/H_2_O, 58/42% *v/v* as mobile phase within 23 min. The developed method was validated in terms of selectivity, linearity, accuracy, and precision.

## 1. Introduction

Dental amalgam has been in use for repairing dental cavities for almost 150 years. However, the Minamata convention for mercury release has affected the use of this traditional material due to concerns over its use and the potential release of mercury intraorally, and the environmental impact during its disposal [1].

Some of their disadvantages of dental fillings with amalgam are their metallic appearance, the high risk of tooth fracture, and the partial removal of tooth structure. There were some controversial studies about the possible mercury leakage, which can cause neuron and kidneys damage and other severe problems regarding human health. However, recent studies conclude that amalgam fillings are safe and should be placed in cases of uncooperative patients and special needs patients or patients with a high risk of caries [2].

However, it is clear from the research that mercury vapor during the removal of amalgam fillings is about ten times more toxic than lead on human neurons and with synergistic toxicity to other metals [3] and environmental concern. Decreasing tolerance of exposure to other mercury compounds will certify that the use of mercury in dentistry remains an issue. Moreover, the Minamata conversion calls for a phase-down approach to dental amalgam (Annex A, Part II) through greater emphasis and research into new dental materials as resin composites [3]. All the above have led to other dental materials, such as methacrylic resins and silicate cement. However, those materials were not as safe as they were expected to be, raising more research in order to establish the biocompatibility and safety of resin composites [4,5].

**Figure 1:**
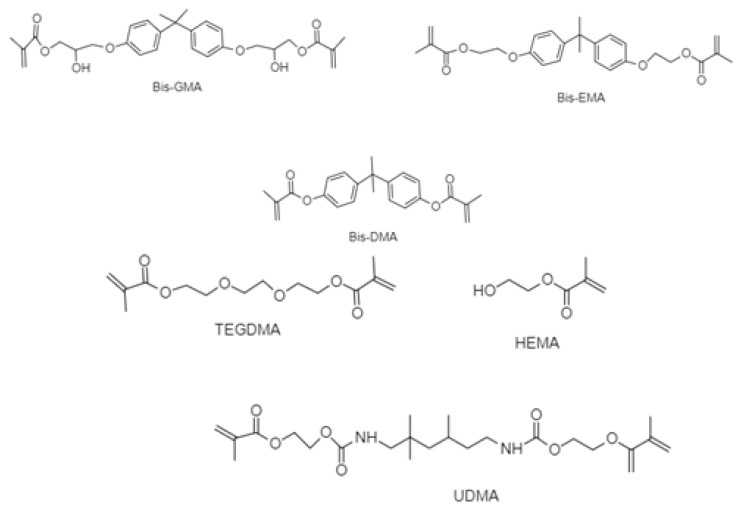
Most commonly used monomers in modern dental materials.

Nowadays, a popular choice among dentists is dental polymer resin-based materials, which consist among others, of a polymeric organic matrix, inorganic fillers (quartz or fused silica, aluminosilicates and borosilicates), and a coupling agent (γ-methacryloxypropyltrimethoxysilane), which is necessary for the chemical bondage between the two [5,6]. Resin composite materials used in preventive and mostly in restorative dentistry have better aesthetic properties, as they are tooth-colored, have better mechanical and compressive strength, and are safer than materials used in the past [4,7]. The organic matrix is derived from organic bisphenol A (BPA)-based monomers, such as bis-GMA (bisphenol-A-glycidyl methacrylate) and its ethoxylated form Bis-EMA, Bis-DMA (bisphenol-A dimethacrylate) and other non-BPA-based monomers, such as TEGDMA (triethylene glycol dimethacrylate), UDMA (urethane dimethacrylate), DMA (N,N dimethylacetamide), and HEMA ((hydroethyl)methacrylate)) [8] (Figure 1).Those monomers are likely to migrate into the oral cavity, and depending on their concentration levels, they can cause allergic reactions, toxic or other effects. The degree of degradation of the composite resins is higher when the polymerization of the organic matrix is not complete, and there are free unbound monomers able to migrate into the immediate environment. The leaching of monomers is also likely to happen due to thermal, chemical, or mechanical factors (e.g., saliva, micro-organisms, chemical ingredients in food, chewing), to which the dental materials are exposed in a daily basis in the oral cavity [9,10].

CAD/CAM technology is a rapid method for the fabrication of dental prosthetic restorations [11]. Commonly used materials that are produced with CAD/CAM are the ceramic-based materials [12]. However, newly introduced materials for use with CAD/CAM technology include resin-composite blocks that are manufactured in high pressure or/and high-temperature polymerization mode and are expected to have exceptional biocompatibility [13], adequate wear resistance [14], superior flexural strength [15], and better internal fit [16] compared to resin composites and ceramic CAD/CAM materials. Moreover, ceramic CAD/CAM materials are more prone to catastrophic and non-reparable fractures than resin-composite CAD/CAM materials [17]. Therefore, resin-composite CAD/CAM materials are preferred by the clinicians for large MOD (mesial–occlusal–distal) cavities than amalgam and from ceramic CAD/CAM materials [18]. However, there is a limited number of studies that have evaluated the leaching of CAD/CAM materials [19]. The organic matrix of some of those materials is composed of BPA-based monomers. BPA-based monomers are endocrine disruptors, and they can interfere with several hormone receptors, causing trouble on the immune system, reproductive organ function both in men and women, the thyroid system and gene expression [20,21]. It is also possible for them to cause oxidative stress, cardiovascular diseases, diabetes, obesity, allergies. Pure BPA is not used during the manufacturing process [22,23]. However, BPA can be found as an impurity or as a degradation product from the hydroxylation of Bis-GMA [24]. Bis-GMA is the most widely used monomer in dental resin materials, and its high viscosity is responsible for the reduced degree of conversion of the monomers, which leads to its higher elution in the human body [25].

The leaching of monomers such as UDMA, TEGDMA may have cytotoxic or allergic effects [13]. In addition, the leaching of those monomers can cause degradation of the material, which will eventually result in the necessity of the replacement of the restoration. Moreover, because several cariogenic bacterial strains can quickly grow and evolve using those monomers as a substrate, secondary caries may evolve. TEGDMA, which is the most frequently used co-monomer, inhibits cell growth and decreases the glutathione level causing trouble in metabolism. It is also genotoxic and interferes with the DNA of the mammalian cells. UDMA is less cytotoxic and has a higher polymerization rate and degree of conversion than other monomers. Nevertheless, it can cause damage to ovary cell DNA, and, in some cases, induct apoptosis [5,25].

In previous years, several liquid chromatographic methods have been developed for the determination of the monomers mentioned above. Some of them study the elution of monomers (mostly BPA, Bis-GMA, TEGDMA, and UDMA) from dental materials after their immersion in methanol or, most frequently, ethanol or deionized water, for a short or an extended period [11,26,27,28,29,30]. Others study the elution from dental resins immersed in artificial saliva [31,32,33]. Only a few high-performance liquid chromatography (HPLC) methods have been developed for the determination of BPA in blood serum or urine [5,34].

The aim of this study is to develop and validate a reliable method to be used as the necessary analytical tool for dentistry to study the respective materials. Thus, a simple, accurate, and precise HPLC method for the simultaneous determination of BPA, Bis-GMA, TEGDMA, and UDMA in artificial saliva is presented herein.

## 2. Materials and Methods

### 2.1. Materials

The CAD/CAM materials were selected according to the specific producer’s classification. Tetric CAD (Ivoclar Vivadent AG, Shaan, Lichtenstein, LOTX29398) has been characterized as a resin-composite CAD/CAM block incorporating an optimized mixture of cross-linked dimethacrylates and inorganic fillers; the material Vita Enamic (Vita, Zahnfabrik, Bad Säckingen Germany, LOT56802) has been the representative of PICN (polymer-infiltrated ceramic network), which incorporates a porous sintered ceramic network filled with plastic. Furthermore, a newly introduced material is produced by filler press, and the monomer infiltration method (FPMI), the only representative of this kind of material, is the Katana Avencia block (Kuraray, Noritake Dental Inc, Hattersheim, Germany, LOT000318). The conventional resin composite Tetric (Ivoclar Vivadent AG, Shaan, Lichtenstein LOTV23649) was selected due to the characterization as a classical resin composite restorative material (Table 1).

### 2.2. Chemicals

Methanol, acetonitrile, and water of HPLC grade were obtained from PanReac AppliChem (Ottoweg, Darmstadt, Germany). BPA, TEGDMA, UDMA, and Bis-GMA were of analytical grade and were supplied by Sigma-Aldrich-LLC (Taufkirchen, Germany). Urea was purchased from Sigma-Aldrich (Steinheim, Germany). NaCl, CaCl_2_, and KH_2_PO_4_ were supplied by Merck KGaA (Darmstadt, Germany).

### 2.3. Artificial Saliva

One liter of artificial saliva consisted of 1 L deionized water, 1 g urea, 0.552 g NaCl, 0.6 g CaCl_2_, and 0.26 g KH_2_PO_4_. Stock solutions of 100 ng/μL of standards were prepared by dissolving the appropriate amount of each monomer in artificial saliva and methanol.

### 2.4. Instrumentation and Chromatographic Conditions

An LC–10 AD pump by Shimadzu (Kyoto, Japan) was used for the delivery of the mobile phase to the analytical column at a flow rate of 1.0 mL/min. Chromatographic separation was achieved isocratically in a PerfectSil Target ODS-3 (250 mm × 4.6 mm, 5 µm) analytical column (MZ AnalysenTechnik, Mainz, Germany) at room temperature, with CH_3_CN/H_2_O, 58/42% *v/v* as mobile phase. The samples were injected via a Rheodyne 7125 injection valve, with a loop of 20 μL volume (Rheodyne, Cotati, CA, USA). Detection was achieved at a wavelength of 215 nm and a sensitivity setting of 0.002 AUFS using an SSI 500 UV–vis detector (SSI, State College, PA, USA).

### 2.5. Preparation of Standards

Stock solutions of 100 ng/μL of BPA, Bis-GMA, TEGDMA, and UDMA were prepared by dissolving the appropriate amount of each monomer in artificial saliva and methanol. Both methanolic and artificial saliva working standards were prepared in the range of 0.1–20 ng/μL for all monomers. Six calibration points were used at concentrations ranging from 0.5 to 20 ng/μL. Standard solutions were stored at 4 °C.

### 2.6. Application to Real Samples

Each CAD/CAM block material was sectioned into 10 × 10 × 2 mm square blocks were prepared using a 0.3-mm thick diamond-coated low-speed band saw (Isomet 1000; Buehler, Lake Blu, IL, USA) under sufficient water coolant. The total number of specimens for each of the CAD/CAM materials and the control material was eighty specimens (N = 80). In the center of each block, a small hole was made by using a 1.5-mm-diameter diamond rotary instrument. Ten specimens of each block were immersed in 8 mL of artificial saliva, and the other ten were immersed in deionized water. A silk thread passed through the hole in the middle of the block, and the specimens were immersed in the solution of each vessel. The glass vessels were airtight closed by using a screw cap. In this way, all surfaces of the specimens could be in direct contact with the medium, ensuring more representative results [35]. All samples were kept at 37 °C. They were measured after 24 h, and 7 s, 14, 28, and 60 days. The immersion solution was refreshed after every measurement.

### 2.7. Sample Size Calculation

The number of specimens for all the groups was defined by statistical calculations. The statistical analyses of the pilot study were carried out using GPower 3.1.9.2 for Mac using the following statistical tests: ANOVA repeated measures within factors, a err prob = 0.05, power (1-b errprob) = 0.80, for three repetitions.

## 3. Results

### 3.1. Chromatography

The separation of the monomers BPA, Bis-GMA, TEGDMA, and UDMA was achieved isocratically with a mobile phase consisting of CH_3_CN/H_2_O (58/42% *v/v*), within 23 min, on an HPLC system with a UV–vis detector set at 215 nm. Retention times were 5.5 min for BPA, 9 min for TEGDMA, 21.5 min for UDMA, and 22.5 min for Bis-GMA. Resolution factors were higher than 3.9, confirming the sufficiency of the separation.

**Figure 2:**
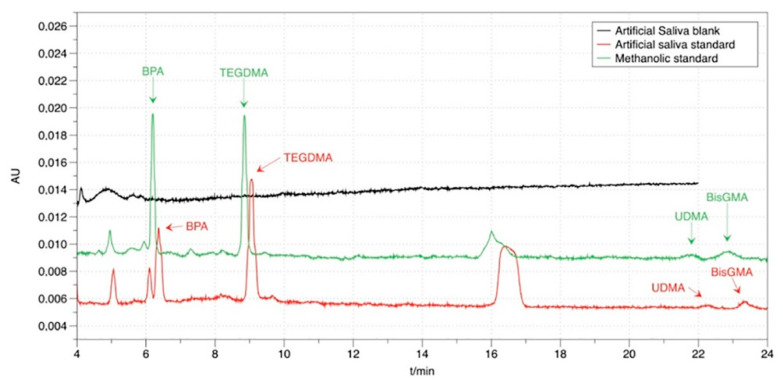
HPLC chromatogram of an artificial saliva blank (black) and an artificial saliva standard solution (red) and a methanolic-standard solution (green).

Typical chromatograms of an artificial saliva blank solution, an artificial saliva standard solution, and a methanolic standard solution are given in Figure 2. The peak at 16 min is probably due to the degradation of one of the monomers, and it was also found in real sample chromatograms. All dental materials analyzed contain TEGDMA and UDMA in their polymeric matrix. In addition to that, the unknown peak is present in all chromatograms of real samples analyzed, containing TEGDMA, and its decrease follows the path of the other monomers. These two facts lead to the hypothesis that it probably is due to the degradation of TEGDMA.

### 3.2. Method Validation

#### 3.2.1. Linearity and Sensitivity

The linearity was evaluated using mixtures of methanolic and artificial saliva standard solutions in different concentration levels within the working range. The upper limit of the linear range was 20 ng/μL for all monomers. Correlation coefficients ranged from 0.991 to 0.997 for artificial saliva solutions and from 0.994 to 0.999 for methanolic solutions. The limit of detection (LOD) and limit of quantification (LOQ) were calculated experimentally with the analysis of standard artificial saliva solutions of concentrations between 0.1 and 0.4 ng/μL. The LOQ was 0.2 ng/μL, and the LOD was 0.06 ng/μL. Calibration curves for each substance investigated were also constructed for standard artificial saliva solutions. The calibration and sensitivity data mentioned above are summarized in Table 2.

#### 3.2.2. Accuracy and Precision

Accuracy and precision were examined at three concentration levels. Mean accuracy was expressed as the recovery (%) of the monomers from artificial saliva standard solutions and was found over 98% in all cases.

Intra-day precision (*n* = 4) revealed RSD% levels below 15%, whereas on concentrations a lot higher than the LOQ RSD% was found under 1%. Inter-day precision measured over a period of five days revealed RSD% values higher than those of the intra-day measurements, but still lower than 15%. The results are summarized in Table 3.

### 3.3. Application to Real Samples

**Figure 3:**
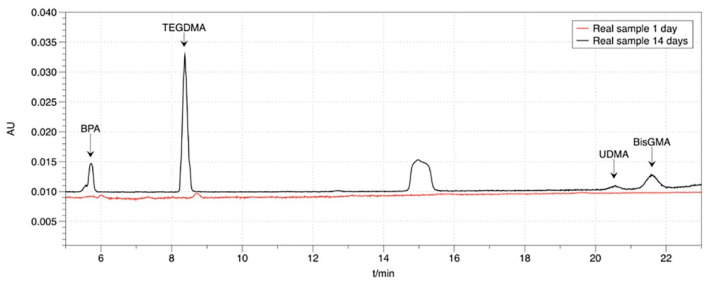
HPLC chromatograms of a conventional resin composite 14 days after its first immersion in artificial saliva (black) and a CAD/CAM composite, 1 day after its first immersion in artificial saliva (red)**.**

Two chromatograms are provided in order to show the applicability of the developed method. A chromatogram obtained from a real sample (conventional resin-composite Tetric) 14 days of immersion in artificial saliva is illustrated in Figure 3. The peak at 16 min is unknown, and its presence is probably due to the degradation of TEGDMA, as mentioned above. For a CAD/CAM material (Vita Enamic), the chromatogram obtained after one day of immersion in artificial saliva is also shown in Figure 3. In this case, the amount of TEGDMA, which is the only monomer released, is small (0.42 ng/μL), so the unknown peak is also present but at an even lower amount.

## 4. Discussion

A simple, accurate, and precise method for the simultaneous determination of BPA, Bis-GMA, UDMA, and TEGDMA from resin-based dental composites in artificial saliva is described herein. For the validation of the developed method, linearity, sensitivity, accuracy, and precision were evaluated. Repeatability and precision, expressed by RSD values, were lower than 15% in most cases. The relative errors ranged from 98% to 106% for artificial saliva standards.

To the authors’ knowledge, there are few articles in the literature to determine the leaching monomers from CAD/CAM materials [19,35]. However, CAD/CAM technology and CAD/CAM materials are beginning to be widely used by the dental community. Composite resins are composed of inorganic or organic fillers embedded in an organic resin matrix composed of Bis-GMA, TEGDMA, Bis-EMA, and UDMA. There are also initiators, stabilizers, and pigments embedded in the composition of those materials. Direct composite resins are applied and polymerized intraorally, but indirect composite resins CAD/CAM millable blocks are manufactured and pre-polymerized extraorally and thus do not hold the disadvantages of direct placement of resin composites such as polymerization shrinkage and inadequate mechanical properties [36].

The lower monomer leaching from CAD/CAM materials, compared to conventional resin-based materials, could be attributed to the different methods of fabrication and the difference of the organic matrix of the materials. The composition of the CAD/CAM materials clearly indicates that the manufacturers have interpolated UDMA and TEGDMA as the preeminent monomer [19]. The advantages of the usage of UDMA include the lower molecular weight of the monomer and the absence of aromatic groups, properties which consecutively decrease monomer release [35]. TEGDMA is a small and agile molecule, which allows the increased conversion of unsaturated double bonds of carbon (C=C) to simple polymer, thus improving the result of polymerization. For the same reason, cross-linking of polymer chains is possible, making the final product more resistant to decomposition by organic solvents. However, the increased degree of conversion is accompanied by a simultaneous increase in polymerization shrinkage, which is one of the main problems of modern composite resin systems [37,38]. However, CAD/CAM resin-based dental materials consist of monomers that can be released in the oral cavity due to the decomposition of the composite. The determination of leaching monomers is essential because those compounds, when transferred into the human body, may cause severe problems, especially in high concentrations. Most of the manufacturers of resin-composite CAD/CAM materials do not mention in the material safety data sheet the exact composition of their product. In this study, the compounds (the name and the CAS number of each dimethacrylate) of the materials that have been chosen were accurately written. There has been a significant amount of research that has evaluated the release of monomers into the oral cavity, and the potential hazardous effects due to monomer release [7] or filler leachability [39] from conventional resin composites, but the research about resin-composite CAD/CAM materials is absent. So, it is necessary to determine the short-term and long-term release of those monomers in order to evaluate the safety of modern dental materials because those materials are subject to hydrolytic effects, which may affect their biocompatibility. The currently presented method is suitable for this purpose, reliable, and requires simple and inexpensive instrumentation. The limitations of this in vitro study that could be addressed in future research include the impact of the oral environment to the leaching pattern of CAD/CAM materials. Variations in the temperature of the oral environment due to food and liquids, the alterations of the pH, and alterations in the flow of saliva could be a protection function concerning the eradication of leaching monomers from resin composite CAD/CAM materials. Another limitation of this study is the unknown peak at 16 min, which is the scope of future research and could not be identified in this research due to limited evidence. Lastly, compared to other studies [5,26,35,40,41], the presented method is more time consuming, as the last monomer (Bis-GMA) was eluted at 22.5 min.

In terms of application to real samples, there was a decreasing pattern for the monomers that were eluted, considering the time intervals for the CAD/CAM materials. This elution stopped completely 28 days after the first immersion of the CAD/CAM materials in artificial saliva or distilled water for all monomers. The most eluted monomer in those materials seems to be TEGDMA. Due to its low molecular mass and its high mobility and activity, its elution from the organic matrix is more likely to happen, compared to the other, less flexible monomers. On the contrary, there was a constant release of all monomers for the conventional resin composite, even 28 days after its first immersion in artificial saliva or distilled water. The above can be attributed to the different methods of fabrication of resin-composite CAD/CAM materials, which consists of high-temperature or/and high-pressure polymerization.

## 5. Conclusions

The herein developed HPLC method is fully validated and proved to be suitable for its purpose as it is very accurate and precise for the newly introduced resin-composite CAD/CAM materials. It requires minimal sample preparation, and the instrumentation involved are inexpensive and straightforward.

## Figures and Tables

**Table 1:** Composition of the materials used.

Material	Type	Composition		Manufacture Lot Number
		Matrix	Fillers	
**ENAMIC**	Polymer Infiltrated Ceramic Material	UDMA, TEGDMA (14 wt %–25 *v/v*)	Feldspar ceramic enriched with aluminium oxide (75% *v/v*), (86 wt %)	Vita Zahnfabrik, H. Rauter GmbH & Co KG, Germany LOT 56802
**AVENCIA**	Nano- ceramic block	UDMA, TEGDMA	SiO_2_ (40 nm)_,_ Al_2_O_3_ (20 nm) (62 wt %)	Kuraray, Noritake Dental Inc., Hattersheim, Germany LOT 000318
**Tetric CAD**	Nano-hybrid block	(28.4 wt %) BisGMA, UDMA, TEGDMA	(64 wt %) barium aluminium silicate glass mean size <1 μm, (7.1 wt %) silicon dioxide with an average particle size of <20 nm.	Ivoclar Vivadent Schaan, Liechtenstein. LOTX29398
**Tetric**	Nano-hybrid composite	(18.8 wt %) BisGMA, TEGDMA, UDMA	Barium glass filler, Ytterbium trifluoride, mixed oxide (63.5 wt %), polymer (17 wt %), additive, catalysts, pigments, stabilizers (0.7 wt %) Particle size: 0.04–3 μm	Ivoclar Vivadent Schaan, Liechtenstein. LOTV23649

**Table 2:** Calibration and sensitivity data of the four examined analytes.

Analytes	Equation	Correlation Coefficient	LOD (ng/μL)	LOQ (ng/μL)
**BPA**	Y = 0.096 × + 0.017	0.997	0.06	0.2
**TEGDMA**	Y = 0.112 × − 0.041	0.998	0.06	0.2
**UDMA**	Y = 0.021 × − 0.017	0.994	0.06	0.2
**Bis-GMA**	Y = 0.065 × − 0.010	0.991	0.06	0.2

**Table 3:** Intra-day and inter-day precision and recovery (%) ± SD of the presented method.

**Monomers**	**Intra-Day** **Precision** **RSD (%) N = 4**			**Recovery (%) ± SD**
	**0.5 ng/μL**	**5 ng/μL**	**10 ng/μL**	
**BPA**	14.2	4.4	0.4	101 ± 5.1
**TEGDMA**	6.7	3.4	0.8	102 ± 22.3
**UDMA**	7.5	6.7	0.1	106 ± 13.1
**Bis-GMA**	15.0	8.1	0.3	98 ± 10.4
**Monomers**	**Inter-Day** **Precision** **RSD (%) N = 3 × 3**			**Recovery (%) ± SD**
	**0.5 ng/μL**	**5 ng/μL**	**10 ng/μL**	
**BPA**	11.9	14.6	3.1	101 ± 5.1
**TEGDMA**	14.4	15.0	0.9	102 ± 22.3
**UDMA**	6.0	14.6	12.3	106 ± 13.1
**Bis-GMA**	3.5	10.4	13.9	98 ± 10.4
